# Comprehensive and quantitative proteomic analyses of zebrafish plasma reveals conserved protein profiles between genders and between zebrafish and human

**DOI:** 10.1038/srep24329

**Published:** 2016-04-13

**Authors:** Caixia Li, Xing Fei Tan, Teck Kwang Lim, Qingsong Lin, Zhiyuan Gong

**Affiliations:** 1 Department of Biological Sciences, National University of Singapore, Singapore 117543.

## Abstract

Omic approaches have been increasingly used in the zebrafish model for holistic understanding of molecular events and mechanisms of tissue functions. However, plasma is rarely used for omic profiling because of the technical challenges in collecting sufficient blood. In this study, we employed two mass spectrometric (MS) approaches for a comprehensive characterization of zebrafish plasma proteome, i.e. conventional shotgun liquid chromatography-tandem mass spectrometry (LC-MS/MS) for an overview study and quantitative SWATH (Sequential Window Acquisition of all THeoretical fragment-ion spectra) for comparison between genders. 959 proteins were identified in the shotgun profiling with estimated concentrations spanning almost five orders of magnitudes. Other than the presence of a few highly abundant female egg yolk precursor proteins (vitellogenins), the proteomic profiles of male and female plasmas were very similar in both number and abundance and there were basically no other highly gender-biased proteins. The types of plasma proteins based on IPA (Ingenuity Pathway Analysis) classification and tissue sources of production were also very similar. Furthermore, the zebrafish plasma proteome shares significant similarities with human plasma proteome, in particular in top abundant proteins including apolipoproteins and complements. Thus, the current study provided a valuable dataset for future evaluation of plasma proteins in zebrafish.

The Zebrafish (*Danio rerio*) has been an increasingly popular vertebrate model not only in developmental biology but also in toxicological and biomedical research. To investigate fundamental molecular mechanisms, omic tools, especially those for transcriptomic analyses, have been increasingly used for profiling important molecules from embryos to various organs such as the liver, kidney, brain, heart, gut, swimbladder, gonads, etc^1^,^2^,^3^,^4^,^5^,^6^,^7^,^8^. However, relatively few proteomic studies have been conducted for zebrafish. Early proteomic studies have mainly relied on gel electrophoresis coupled with tandem mass spectrometry analysis (MS/MS) and zebrafish proteomes was reported for embryonic stages^9^,^10^,^11^, liver^12^ and lens^13^. The number of proteins identified is quite limited, ranging from less than a hundred to about two hundreds. In the past five years, liquid chromatography (LC) for protein separation before MS/MS has been increasingly used. With this approach, zebrafish proteomes have been reported for multiple organs, including gills^14^, brain^15^, olfactory bulb^16^, fin^17^, skeletal muscle^18^, etc. The number of proteins identified with the new technology varies from a few hundreds to over five thousand. In recent years, there is also an increasing trend to apply proteomics in zebrafish for developmental analyses, disease studies and assessments of chemical toxicity.

Unlike other tissues and organs, zebrafish blood and plasma are rarely used for omic studies. Most studies on zebrafish blood aimed at hematopoiesis, immunity, hemostasis and thrombosis, and hematologic disorders through genetic manipulation, biochemical and imaging analyses^19^. Other blood studies included measurement of hematological parameters and assays for specific components as a monitoring tool for abnormal conditions, e.g. cholesterol and triglycerides in plasma for lipid accumulation in arteries^20^ and blood glucose for pancreas function^21^. However, taking into account the growing interest in the zebrafish models and the great potential of plasma biomarker discovery, there is an urgent need to understand the basic plasma protein profile.

Plasma is the most commonly used clinical sample and there have been increasing numbers of studies in pursuing plasma/serum biomarkers for various diseases, for it contains not only blood proteins but also proteins secreted from almost all tissues. Plasma Proteome Database (PPD), an online resource for human plasma proteins, was developed in 2005 as an effort to characterize human plasma proteome. By 2014, it has been updated with over 10,000 reported plasma proteins based on more than 500 studies^22^. The characterizaztion of mouse plasma proteme has also been conducted and Zhang *et al.* have published a repository composed of 568 LC-MS/MS runs, which contains over 3000 mouse plasma proteins with abundance spanning seven orders of magnituds^23^. After immunodepletion of highly abundant proteins prior to LC-MS/MS, Lai *et al.* have detected 4,727 mouse plasma proteins that span the range of concentrations from 10 pg/mL to 2.65 mg/mL^24^. In contrast, studies of zebrafish plasma remain rather limited, partly due to the technical difficulties in blood sampling and the small volume of blood available from each fish. The amount of blood collected from each adult fish varies between 1–10 μL^25^,^26^,^27^,^28^. Volume of plasma collectable is typically 40–50% of whole blood. With the minute amount of plasma, two research groups have applied LC-MS/MS to profile the proteome: one group has detected about 150 proteins in a pooled sample from two fish^17^ and the other has detected about 130 proteins from six individual fish^27^. Neither study has attedmpted to estimate the concentration of individual proteins identified. These numbers are far below those commonly identified from mammalian plasma, which generally range from several hundreds to over a thousand proteins^29^, with the highest number of 9,087 proteins identified in a single study^30^.

Thus, with the current advances of proteomic technology, it is necessary to have a more comprehensive examination of proteomic profiles of fish plasma and the information should be valuable for future plasma related research in fish, in particular in discovery of non-invasive biomarkers for diseases and chemical toxicity. In this study, we employed two MS approaches to plasma samples collected from both male and female zebrafish: first profiled by conventional shotgun LC-MS/MS for an overview of the proteome and then followed by a quantitatively analytical method called Sequential Window Acquisition of all THeoretical fragment-ion spectra (SWATH)^31^ to examine proteins with differential abundance between two genders. We have identified a total of 959 proteins with estimated concentrations ranging from 0.9 µg/mL to 8.6 mg/mL. 35 of these proteins are significantly gender-biased in the SWATH profile.

## Results and Discussion

### Abundance and distribution of zebrafish plasma proteins

Methods of blood collection from zebrafish mainly include tail ablation, heart puncture and dorsal aorta cut. After testing these methods, we chose a modified tail ablation method for its short processing time and reproducibly high yield. Using this method, 5–10 μL of blood was routinely collected from male fish and 8–15 μL from female fish. Occasionally up to 12 μL was collected from a male and 20 μL from a female. Plasma was analyzed using 10% SDS-PAGE for quick assessment of protein abundance patterns. Silver staining of individual fish plasma revealed high similarity within the same gender as well as divergence between genders (Supplementary Fig. S1). The protein patterns of plasma collected from 6-month-old fish were similar to those from 1.5-year-old fish. Plasma protein concentration was generally lower in males (37.7 ± 8.4 mg/mL) than in female (53.1 ± 9.3 mg/mL), with *p* < 0.011 in two-tailed heteroscedastic *t*-test. Unlike human plasma where the albumin constitutes at least half of total proteins and forms predominantly large band in gels, the zebrafish does not have albumin^32^ and had no overwhelmingly strong protein bands in gel electrophoresis of plasma proteins, except for two bands of about 150 kDa (putatively vitelogenins) in females.

For comprehensive proteomic profiling of male and female zebrafish plasma, fresh plasma of nine individuals of each gender were pooled. The total protein concentrations of plasma were 34.6 mg/mL for the male sample and 49.8 mg/mL for the female sample. An aliquot of 2 μg of total plasma protein was used for MS analysis without depletion or enrichment. Six technical replicates of shotgun MS were performed to increase the coverage of proteins. Unique proteins were identified based on various numbers of significant peptides, among which about 40% were identified with two or more significant peptide hits (Supplementary Fig. S2).

**Figure 1:**
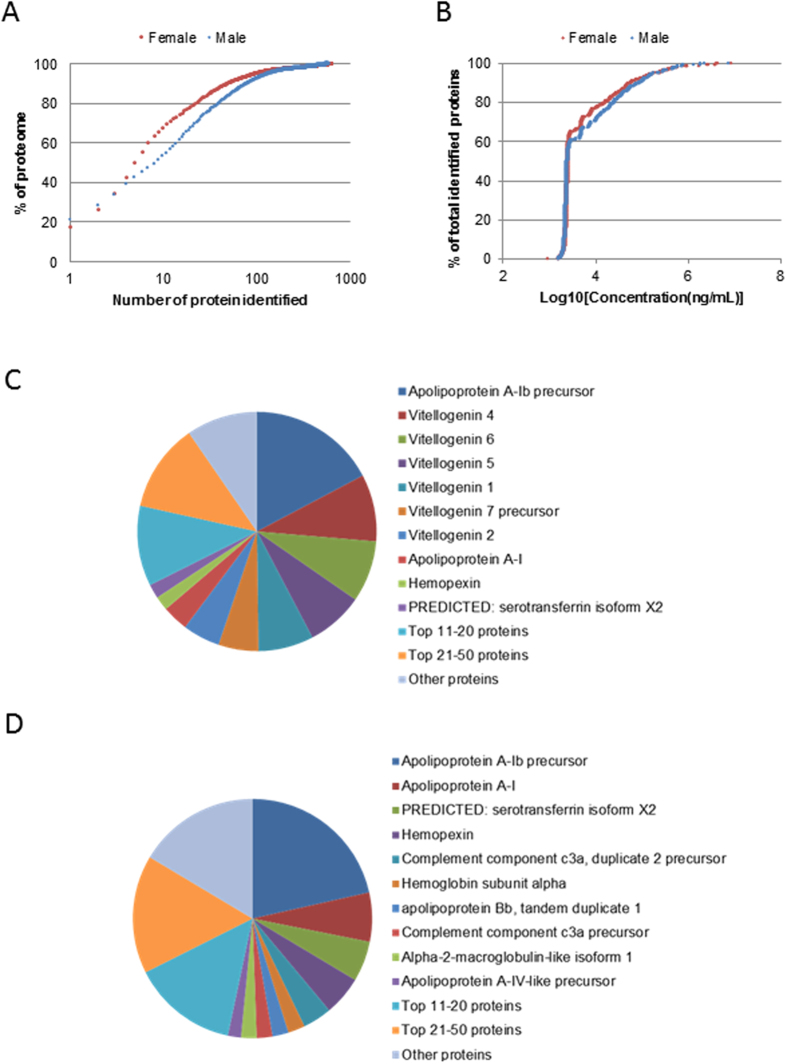
Overview of plasma protein profiles based on shotgun MS. (**A**) Cumulative percentage of plasma proteome ranked according to protein abundance. (**B**) Cumulative plasma protein numbers (shown as % of total identified proteins, 642 in female and 595 in male) ranked based on estimated concentrations of proteins detected. Most proteins (~70%) were in the range of 2–10 μg/mL. (**C,D**) Abundance distribution of plasma proteins in females (**C**) and in males (**D**). Top 10 proteins account for about 65% of plasma proteins in female and about 50% in male.

A total of 666 protein forms were identified in female plasma and 624 in male plasma, resulting in 959 unique proteins in total, 289 of which were common in both genders. 949 unique proteins (642 in female and 594 in male) had semi-quantitative information (Supplementary Tables S1 and S2). Concentrations of individual proteins were estimated using the total protein concentration, molecular weight of the proteins, and their corresponding emPAI scores in the shotgun profiles. Distribution patterns of molecular weights for female and male plasma proteins were similar (Supplementary Fig. S3A). The number of top abundant proteins contributing to 90% of plasma proteins was smaller in female (49) than in male (80) (Fig. 1A). Proteins detected in female plasma ranged from 0.9 μg/mL to 8.6 mg/mL. Similar range of protein concentration distribution (1.6 μg/mL to 7.4 mg/mL) was also found in male plasma. For both genders, most proteins were 2–10 μg/mL (Fig. 1B). Highly abundant proteins (>100 μg/mL) were essentially the top 50 proteins (54 in female and 49 in male). Proteins of intermediate concentrations (10–100 μg/mL) included various serine protease inhibitors (Serpins), complement factors and components, coagulation factors, and some metabolic enzymes (e.g, Muscle creatine kinase a, Carboxylesterase 3, Glyceraldehyde-3-phosphate dehydrogenase, Aldolase a, Glutathione peroxidase 4 and beta-enolase). In this study, we also detected a few low-abundant proteins (<2 μg/mL), including Eukaryotic translation elongation factor 2b, Retinol-binding protein 3, Semaphorin-5B and Catenin delta-2.

Female plasma contained abundant vitellogenins (Vtgs) which were not detected in the male shotgun profile. Vtgs are egg yolk precursor proteins and all seven known Vtgs in zebrafish^33^ were detected in this study. Six of them (Vtg1, 2, 4, 5, 6 and 7) were among the top 10 abundant proteins, while Vtg3, whose gene is located on a different chromosome from other Vtg genes^33^, was ranked 18 (Table 1). Among these Vtgs, Vtg3, 4, 5, 6, and 7 had molecular weight of ~150 kDa and probably together (~31% of total plasma protein mass) gave rise to the most abundant band in the SDS PAGE of female plasma (Supplementary Fig. S1), while Vtg1 (~7, 0.5% of total plasma protein mass), with a molecular size of 211 kDa, probably accounted for the prominent band of ~200 kDa. However, if Vtgs were excluded, both female and male plasma had similarly abundance in apolipoproteins and the top 30 abundant proteins largely overlapped (22/23) (Table 1). In both genders, the most abundant protein was Apoliproprotein A-Ib, accounting for about 20% of total plasma proteins detected. Other common abundant proteins included various apolipoproteins, hemopexin (Hpx), complement component C3, hemoglobin subunits, and fibrinogen alpha and beta chains. Top 10 abundant proteins constituted 67.6% of plasma proteins in female and 53.3% in male (Fig. 1C,D).

We compared the 959 plasma proteins detected with those reported in the two published studies^17^,^27^ and found that most proteins (820/959) in this study were newly identified in zebrafish plasma (Supplementary Fig. S3B). About 80% of these newly identified proteins were below 7.5 μg/mL and 95% below 50 μg/mL. Based on the concentrations estimated, we detected proteins across almost five orders of magnitude without depletion of abundant proteins or enrichement of low-abundant proteins.

### Characteristics of plasma protein composition

**Figure 2:**
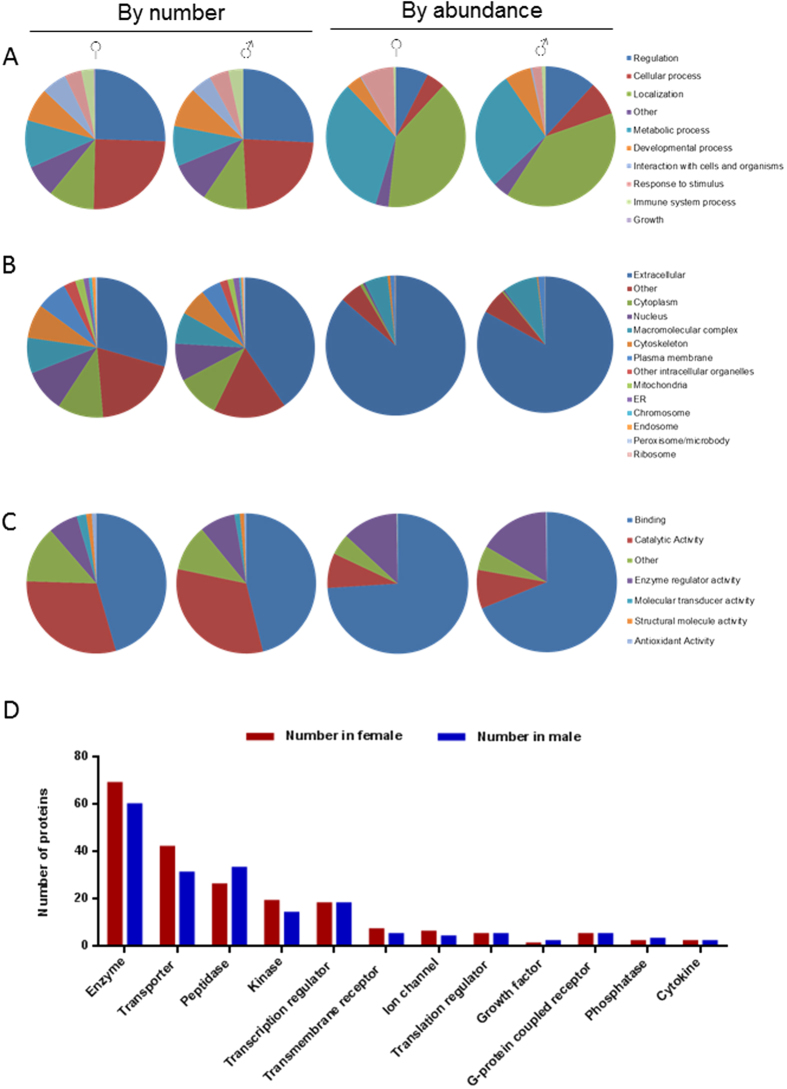
Characteristics of plasma protein composition. (**A**–**C**) GO distribution of plasma proteins based on number of protein entries (left) and protein abundance (right) in both genders as indicated. GO annotation was retrieved for a total of 791 unique UniProt entries of zebrafish plasma proteins. Categories of GO are: Biological Process (**A**), Cellular Component (**B**) and Molecular Function (**C**). (**D**) Comparison of types of plasma proteins between the two genders as classified by IPA.

To obtain an overview of the biological functions of these plasma proteins, Gene ontology (GO) annotations were retrieved using software STRAP. Combining proteins detected in both genders, a total of 795 Uniprot IDs were obtained. GO distributions for Biological Process, Cellular Component and Molecular Function are presented in Fig. 2A–C. Female and male plasma shared very similar distribution patterns. Top terms in Biological Process were Regulation (~25%), Cellular process (~25%), Localization (~10%), Metabolic process (~10%) and Developmental process (~10%). However, in term of protein abundance, Localization (~30% in female and ~40% in male) and Metabolic process (~25% in female and ~35% in male) were the two largest categories. For Cellular Component, the top term was Extracellular (~30% in female and 40% in male). Interestingly, a significant portion of the proteins was found to be in the cytoplasm (~10%), nucleus (~10%), and plasma membrane (~7–8%), while a small portion was from mitochondria, chromosome, endoplamic reticulum and endosome (~0.8–2%). These indicate that zebrafish plasma contained many cellular proteins that probably leaked from different tissues. As expected, in term of protein abundance, Extracellular proteins are predominant in both females (~40%) and males (~60%). For Molecular Function, most plasma proteins fell in the categories of Binding (~45%) and Catalytic activity (~30%), with a small percentage of proteins in the category of Enzyme regulator activity (~7% in female and 8% in male), Molecular transducer activity (~2% in female and ~1% in male) and Antioxidant activity (~1% in female and 0.5% in male). If protein abundance is taken into consideration, higher protein contents were attributed to Binding for Molecular function (Fig. 2C).

To further understand the composition of plasma proteins, we mapped the 959 total proteins with corresponding zebrafish Gene IDs in NCBI database and input 910 mapped IDs to IPA to retrieve annotation on types of proteins. Annotation for 518 plasma proteins (57% was retrieved and the composition was similar in both genders (Fig. 2D and Supplementary Table S2). Based on number of proteins belonging to each defined type, the largest category was Enzyme, followed by Transporters, Peptidase, Kinase and Transcription regulator. However, in term of total weight fraction in plasma, enzymes account for only 1.7% in female and 3.9% in male, while transporters account for 33% in female and 54% in male. These transporters include macroglobins, apolipoproteins, transferrin and hemoglobins. Notably, two growth factors (angiotensinogen and Hepatocyte growth factor-like protein) and two cytokines (Complement component 5 and Interleukin-27 subunit beta) were also detected, which ranged from 2.3 μg/mL to 222.0 μg/mL. There were 242 IDs mapped to the term “Others”, yet accounting for only about 5.1% protein content in female plasma and 9.5% in male.

### Tissues expressing plasma proteins

**Figure 3:**
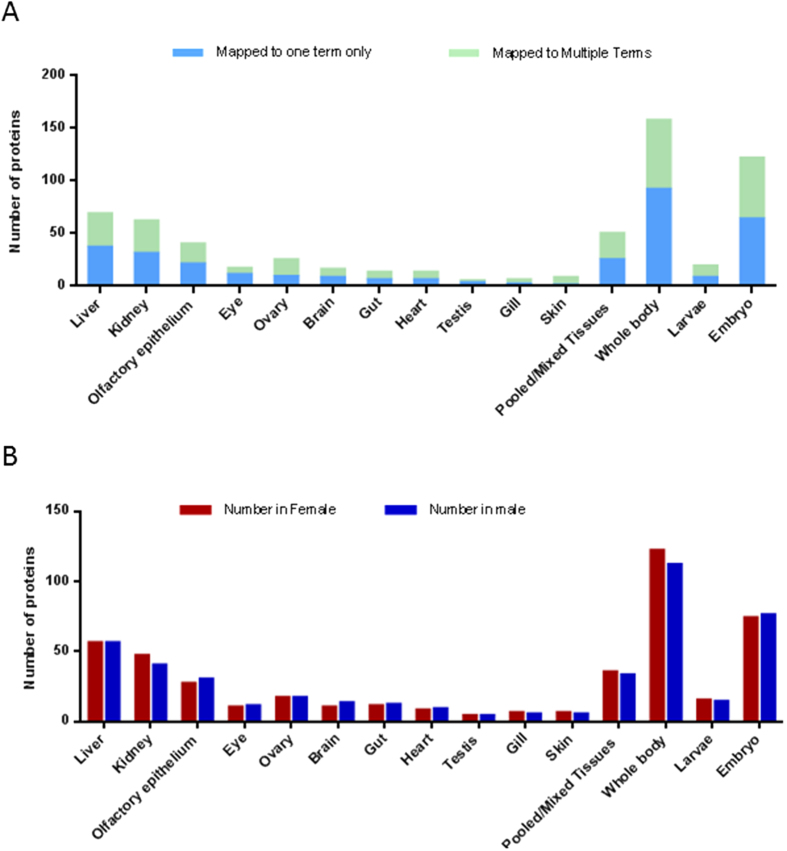
Tissue expression of plasma proteins. (**A**) Distribution of plasma proteins based on tissues of expression. (**B**) Comparison of tissue expression of plasma proteins between females and males. Only those proteins expressed in a single tissue are used for comparison.

To determine which organs are major contributors to plasma proteins, the 910 Gene IDs were input to DAVID database for information on tissue expression. Information on tissue expression was retrieved for 452 genes (Supplementary Table S3). Most of the proteins were found to be expressed in whole body or in multiple organs (Fig. 3A). 135 genes had reported expression in only one individual organ, including liver (37), kidney (31), olfactory epithelium (21), eye (11), ovary (9) and brain (8) (blue bars in Fig. 3A). If the abundance of proteins is taken into account, the liver is the single predominant site of production for plasma proteins, with 37 proteins accounting for about 30% of total plasma protein content in both genders (Supplementary Table S3). These 37 proteins included high abundance proteins such as Vtgs and some complement components. There were also other abundant proteins that were expressed in both the liver and other organs, such as apolipoproteins. In fact, according to our in-house zebrafish liver transcriptome generated by RNA sequencing, mRNAs for at least 323 plasma proteins detected in the present study are also expressed in the liver, among which Vtg mRNAs take up as much as 78% of female liver transcriptome^6^. The extremely high expression of Vtgs in the liver leads to the high abundance of total Vtgs (>40%) in the plasma of female. Since the expression level of liver secreted proteins is generally in agreement with corresponding plasma protein concentrations^34^, alteration of liver functions could result in prominent changes in plasma proteome. Unlike those from liver, proteins from other organs are present at low concentrations (Supplementary Table S3). For example, 31 proteins from kidney only make up about 1–2% of total mass, while 21 proteins from olfactory epithelium only make up 0.1%.

### Comparison of zebrafish and human plasma proteome

The range of human total plasma protein concentration is 60–80 mg/mL, with concentrations of detected proteins spanning at least 12 orders of magnitudes^35^. As compared to human, the total plasma protein concentration seems to be lower in zebrafish (30–55 mg/mL), with females often having higher total protein content than males.

To compare the zebrafish plasma proteins with those reported in human, we searched against NCBI database and mapped 882 out of the 959 zebrafish plasma proteins to 598 unique human homologs. These human homologs were examined against PPD (http://www.plasmaproteomedatabase.org/). Most of these proteins (435/598, or 73%) have been detected in human plasma and 140 of them have concentration values available. In comparison with human plasma, zebrafish plasma has at least three prominent differences: the absence of albumin, the presence of vitellogenins, and the presence of multiple homologs of the same human protein. For example, in zebrafish plasma, there are at least three homologs of human Apolipoprotein A and seven homologs of Alpha-2-macroglobulin. In terms of protein composition, human albumin comprises approximately 50% of the total plasma content and the top 22 proteins make up 99% of the total proteins^30^. In zebrafish, the highly abundant proteins take up a smaller percentage. The top most abundant protein, Apolipoprotein A-Ib precursor, comprises approximately 20% of total proteins detected. Furthermore, it takes at least top 50 proteins to account for 90% of the total content and top 400 proteins for 99% content (Supplementary Tables S1 and S2).

**Figure 4:**
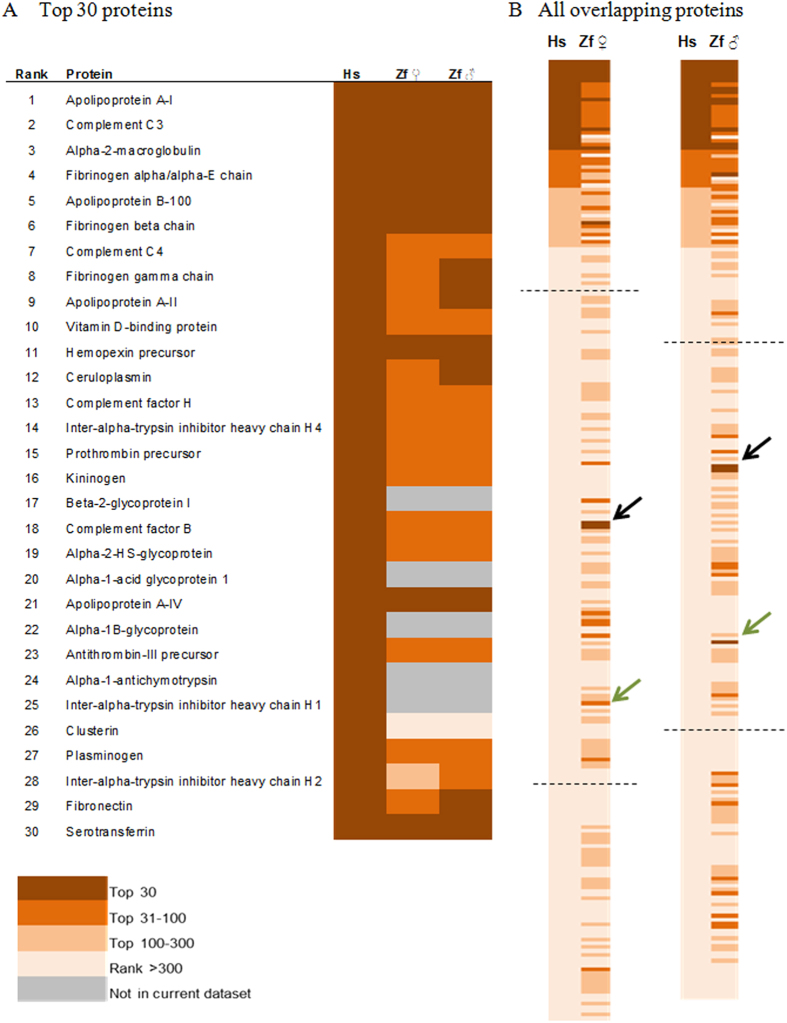
Overlaps in ranks of plasma proteins between human and zebrafish based on the study of Liu***et al.***^36^. (**A**) Rank of top 30 abundant human plasma proteins in zebrafish. (**B**) Comparison in ranks of all mapped overlapping plasma proteins between human and zebrafish female (left, 255 proteins) and male (right, 250 proteins). Proteins are presented based on ranks in human plasma. Two dashed lines in (**B**) mark approximately the rank of 500 and 5000 in human plasma proteins. Hs: human; Zf: zebrafish. Black arrows indicate Alpha-1-antitrypsin precursor (human) and Serpina1 protein (Zebrafish), and green arrows indicate Ig mu chain C region.

To compare the abundance ranking of plasma proteins between zebrafish and human homologs, we mapped the human homologs of detected zebrafish plasma proteins to those reported by Liu *et al.*^36^. Among the top 30 most abundant proteins identified in human after depletion of several highly abundant proteins, we found that at least 13 of them, such as Apolipoprotein A-I, Complement C3, Alpha-2-macroglobulin, alpha and beta Fibronogens, Apolipoprotein B-100, etc., were also among the top 30 abundant proteins in zebrafish (Fig. 4A). Essentially all the top 30 serum proteins in human (except for four proteins that were not identified in zebrafish) were among the top 100 zebrafish serum proteins and these proteins were enriched with apolipoproteins and complement proteins. Thus, there are similar profiles of abundant plasma protein with conserved main functions in fatty acid transport and immunity between human and zebrafish. When the ranks of all overlapping plasma proteins were compared between human and zebrafish (Fig. 4B), we found that there were higher similarities in the high (top 100) and medium ranks (top 500) for both male and female zebrafish but much lower similarities in the low ranks. We speculate that, due to the much smaller coverage in zebrafish with non-depleted plasma (~1,000 proteins) as compared to the large profile (~9,000 proteins) of immune-depleted human plasma, concentration for zebrafish proteins were generally over-estimated and the effect was much more significant for those of lower ranks. It is interesting to note that some top abundant zebrafish proteins correspond to low abundant protein in human, such as Alpha-1-antitrypsin precursor and Ig mu chain C region (Fig. 4B); this is apparently due to immunodepletion of abundant proteins (including antitrypsin and immunoglobins) in human samples for enriching low abundant proteins.

**Figure 5:**
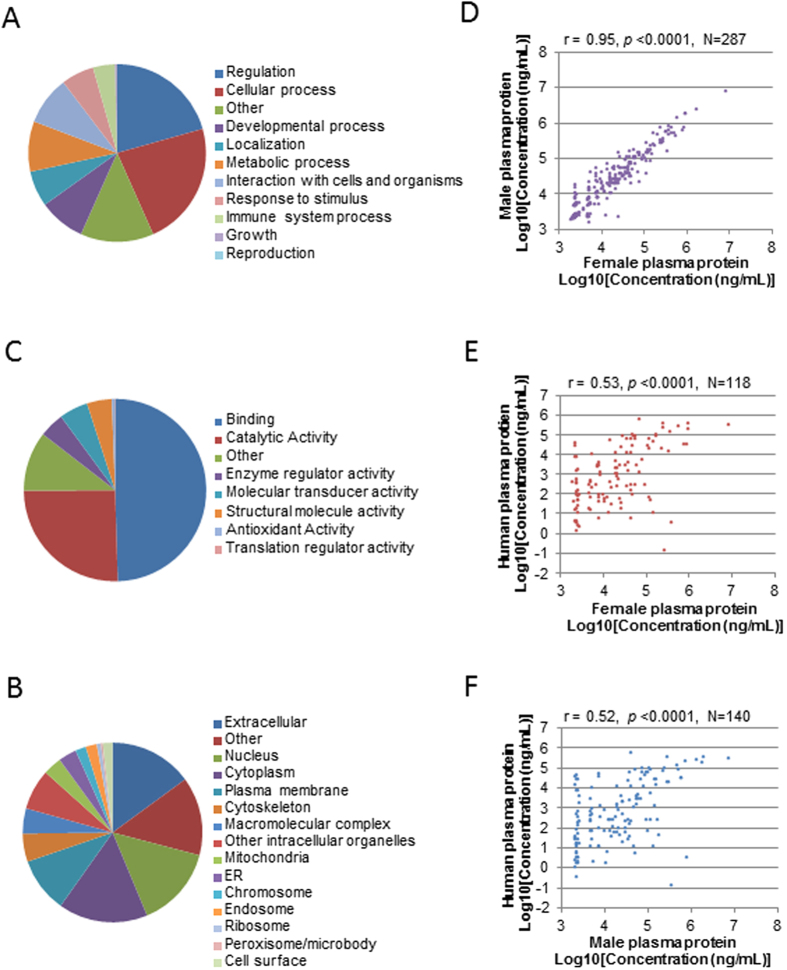
Comparison of zebrafish and human plasma proteomes based on Plasma Proteme Database (PPD). (**A**–**C**) GO distribution of human plasma proteins in the categories of Biological Process (**A**), Cellular Component (**B**), and Molecular Function (**C**). Both the ranks of terms and the corresponding percentages are similar to those in zebrafish plasma (Fig. 2A–C). (**D**) Pearson correlation between zebrafish female and male plasma proteins based on concentration of overlapping proteins. (**E**,**F**) Pearson correlation between human plasma proteins and zebrafish female (**E**) and male (**F**) plasma proteins, based on corresponding concentrations of homologous proteins.

To compare the distribution of GO annotation terms between zebrafish and human plasma, through PPD, we downloaded proteins that have been reported in at least two human plasma studies and obtained a list of 3,638 unique UniProtKB accessions. GO annotation of these proteins was retrieved using STRAP and plotted (Fig. 5A–C). As compared to zebrafish plasma (Fig. 2A–C), for both Biological process and Molecular function, top two terms were the same and the corresponding percentages are also similar. Terms at lower ranks overlapped between the two species with both similarities and differences in percentages. As for Cellular component, more human plasma proteins fell under the terms such as “cytoplasm”, “nucleus” and “plasma membrane”, while higher percentages of zebrafish proteins belong to “extracellular” and “others” (Figs 5B and 2B). This is likely due to two reasons: (1) better GO annotation is available for human than zebrafish; (2) the coverage of human plasma proteins from a composite database is much higher than our single study, such that the human dataset contains more low-abundant proteins secreted or leaked from cellular compartments, which were not detected in this study.

To test whether there is a direct correlation in corresponding plasma proteins, we compared the estimated concentrations of zebrafish proteins with published concentration values of their human homologs and performed Pearson correlation test (Fig. 4D–F). While high correlation was observed between zebrafish genders (r = 0.95), correlation across two species were much lower. Yet, decent positive correlation was still observed between human and both zebrafish genders (*p* < 0.0001), with a correlation score of 0.53 for female and 0.52 for male. The detection limit of this study was apparent in the correlation plots, which might have compromised the accuracy of concentration estimation for low-abundant proteins and thus gave rise to an under-estimated correlation score between the two species.

The major organ for synthesis of human plasma proteins is the liver and at least 362 plasma proteins of liver origin have been reported^37^. Liver diseases are often accompanied with manifestations in hematological disorders^38^; thus, routine clinical screening of functional status of liver are generally based on amount and activity of selected enzymes of liver origin in serum samples^37^. Similarly, in the present zebrafish plasma proteome, at least 323 plasma proteins whose transcripts were also found in zebrafish liver^39^. These include proteins whose human homologs are liver disease markers; for example, apolipoprotein E and M, AHSG, and complement C3 are biomarkers for hepatocellular carcinoma (HCC)^40^. In human, plasma biomarkers for disease status of other organs have also been studied and homologs of a few such biomarkers (putative or in use) are also present in zebrafish plasma. For example, biomarkers for ovarian cancer (apolipoprotein A-I and transferrin)^41^, Lung cancer (LDH and haptoglobin)^42^,^43^, and pancreatic cancer (Plasminogen, Hemopexin, Complement factor H, and alpha-2 macroglobulin)^44^.

### Gender-biased plasma proteins

Sexual dimorphism in gene expression pattern has long been observed in human and there is increasing evidence on the differential risks and responses to diseases due to gender disparity^45^. In terms of human plasma proteins, the gender-associated differences between healthy subjects in general are not well studied. Based on a small-scale proteomic study of five men and five women, five female-biased proteins (pregnancy zone Protein, coagulation factor V, α_1_-antitrypsin, β_2_-microglobulin, and complement factors H) and five male-biased proteins (Fc binding protein, protein Z-dependent protease inhibitor, phosphatidylinositol-glycan specific phospholipase, protein S-100 and transgelin-2) have been identified^46^. A few more studies focusing on the concentration difference of a specific protein or few related proteins as well as the consequence of such difference under a disease condition have been reported. For example, the sex-differential abundances of candidate proteomic markers in cardiovascular diseases^47^ and in non-small cell lung cancer and asthma^48^ have been reported.

In this study, SWATH was employed to investigate the difference between male and female zebrafish plasma. SWATH MS is a data-independent acquisition method which combines the advantages of high throughput shotgun proteomics and quantitative MRM (multiple reaction monitoring) approach. Through systematical fragmentation of all precursors in a moving mass window, a complete record of all detectable precursors and their MS/MS ion fragments is generated. This allow for the identification of proteins via matching with a pre-generated spectra ion library and MRM-like quantitation of identified proteins^31^. An ion spectra library was built from the shotgun profiling and triplicate runs were then performed in SWATH mode for quantitative comparison of individual plasma proteins between two genders. There was a good correlation between technical triplicates (Supplementary Fig. S5A,B). A total of 200 proteins were quantified for both male and female plasma samples with high confidence (Supplementary Table S5), which include most abundant proteins as well as some relatively low-abundant proteins such as Nothepsin (7.8 μg/mL) and Vitelline membrane outer layer 1 homolog b (2.4 μg/mL). Not surprisingly, variation between replicates was smaller for proteins of high abundance than those of lower abundance.

**Figure 6:**
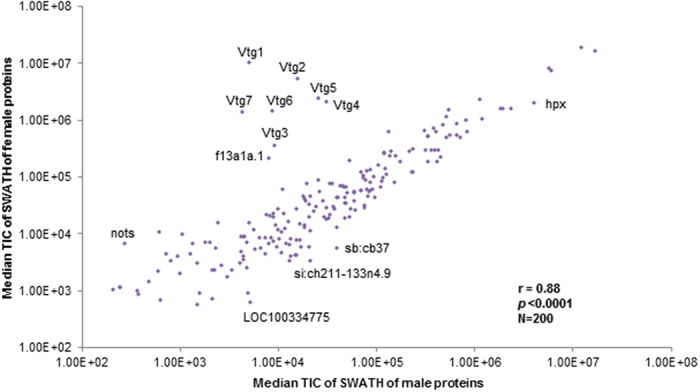
Overview of SWATH profile of female and male plasma proteins depicting abundance of detected proteins. Median total ion current (TIC) of proteins identified in male (x-axis) and female (y-axis) plasma were compared. Majority of plasma protein have similar abundance in both sexes (Pearson correlation = 0.88), with representative sex-biased plasma proteins as outliers.

Most proteins were of similar abundance across genders and only a few proteins showed prominent differences between genders, among which Vtgs were the most obvious ones (Fig. 6). To identify gender-biased proteins, the average total ion current (TIC) values for each protein were compared and 35 differential proteins were obtained based on two criteria: *p* < 0.05 and FC > 2 (Table 2). Quantitation of these proteins in shotgun profiles was mostly consistent (Pearson correlation r = 0.76), except that some proteins of lower concentrations (mostly <10 μg/mL) were either not detected or with inconsistent quantitation (Supplementary Table S6). Based on cross-gender difference smaller than 5% and small coefficient of variation (CV) in SWATH profile, we also identified apolipoprotein A1 (Apoa1) as a stable protein across genders and technical replications. It has only 2% difference between genders (FC = 0.98) and a small CV (12.8% in female and 6.6% in male) in SWATH profile. It was among the top 10 proteins in both genders according to the shotgun profile (~2 mg/L). In comparison, beta-actin, the popularly used as a loading control in western blot of zebrafish plasma^49^,^50^, had a moderate concentration of ~20 μg/L in both genders in shotgun profile. In the SWATH profile, beta-actin showed 8% difference (FC = 1.12) between the two genders and much greater CVs (29.5% in female and 32.4% in male). Thus, in addition to beta-actin, Apoa1 could be a potential reference protein in zebrafish plasma studies for normalization of quantification of other proteins.

Among the 26 female-biased proteins, all seven vitellogenins (Vtg1 to Vtg7) were among the top with high fold changes ranging from 20 (Vtg2) to 1552 (Vtg1) fold. Another ovary-associated protein, Vitelline membrane outer layer 1 homolog a (Vmo1a), was also 6.4 fold more abundant than that in male. Two other proteins, Nothepsin and an uncharacterized homolog of human Coagulation factor XIII (FXIII), showed about 23 fold changes. In addition, apolipoprotein E related proteins (Apolipoprotein Eb and Apolipoprotein E precursor) were also 4.2 and 3.5 fold more abundant in female. Other female-biased proteins included Phosphoglycerate kinase 1 (Pgk1), a predicted keratin 8 homolog, and Muscle creatine kinase a and b (Ckma and Ckmb), and a few other apolipoproteins. The fold changes of the 9 male-biased plasma proteins were much smaller. A Carboxylesterase 2-like protein showed the greatest fold change of 3.6, followed by Ependymin (3.4 fold) and Serpina 1 protein like (3.1 fold). Other male-biased proteins include Sex hormone binding globulin (Shbg), a predicted complement C4-B, Myoglobin and uncharacterized proteins.

As compared to a previous study^27^, only the Vtgs have been earlier reported as gender-biased proteins, while the rest were newly found in this study. As there have not been many studies on the sexual dimorphism of plasma proteins, only few of these 35 proteins have been shown to have gender-difference in plasma or other tissues. In zebrafish, *vtg* genes are highly expressed in the liver of female fish and estrogen-treated male fish^6^,^33^. In females, Vtgs were transported in blood and taken up by oocytes,, whereas in males, they remain in blood until degradation^51^. The induction of Vtgs in the liver and plasma of male fish have been widely used as molecular markers for environmental estrogens^6^,^51^. Other than the Vtgs^27^, Nothepsin, a paralog of Cathepsin, has been reported to express only in the liver of female zebrafish^52^, though the protein has also been detected in the ovary^53^. The gene for Nothepsin has been reported to be among the top 20 most regulated genes during vitellogenesis in female liver and after E2 treatment of male liver^54^. In human, Cathepsin D is a secreted protein from various types of cancer cells and its serum level has been proposed as a biomarker for breast cancer and a few other cancers^55^. Coagulation factor XIII (FXIII) circulates in human plasma and is involved in the formation of fibrin structure as well as regulation of fibrinolysis; in zebrafish, this female-biased homologous protein, named FXIIIa-42, has not been well studied but it has been reported to be upregulated prior to vertebral calcification of larvae^56^. Gender-biased expression of FXIII in human or zebrafish has not been reported before; however, in a transcriptomic analyses of gene targets of estrogen, *f13a1a* (coding gene for FXIIIa-42) was highly induced by E2 in both embryos and male adults^57^. In addition, in human, FXIII also plays a role in maintaining pregnancy^58^. All this suggests that FXIIIa-42 may also play important roles in females. Another interesting female-biased protein is Pgk1, which is an erythrocyte glycolytic enzyme gene located on X chromosome in human; mutation of this gene causes non-spherocytic haemolytic anemia and consequently various neurological disorders^59^.

The top male-biased protein Carboxylesterase 2-like (Ces2), is a homolog of human CES1. The human plasma CES1 has been proposed to be a serological marker candidate for HCC and it has performed better in discriminating HCC from other liver diseases in a recent clinical study^60^. It is interesting to note that Ces2-like protein is male-biased in zebrafish plasma, a fact consistent with the high HCC incidence in men^61^. Another noteworthy male-biased protein is Shbg. In human, SHBG is a major plasma transporter of sex steroids and is associated with insulin resistance; however, the concentration of SHBG tends to be higher in women than men^62^. This difference may indicate functional variation of SHBG between human and fish.

As the liver is the major organ for plasma protein production, it is also of interest to compare the gender-biased plasma proteins with differentially expressed genes between male and female livers. We have previously reported sexual dimorphism of gene expression in zebrafish liver and relevant effects of sex hormones^6^. Consistent with the highly female-biased abundance of Vtgs and Nothepsin in female plasma, gene expression of *vtgs* and *nots* was highly enriched in liver of naïve female and E2-induced male but down-regulated in KT11-treated female. An uncharacterized male-biased protein, si:ch1073-126c3.2, also showed male-biased gene expression in liver and it was down-regulated in E2-treated male. Interestingly, this protein was also down-regulated in KT11-treated male but not affected by either hormone treatment in female. Another gene *shbg*, coding for male-biased plasma protein Shbg, also showed 10-fold enrichment in the control male liver as compared to female. Different from what was observed in plasma proteins, gene expression for four female-biased apolipoproteins, Apobb.1, Apoc2, Apoea and Apoeb, were 3–9 fold higher in control male and not deregulated by either hormone in either gender. However, it should be noted that these proteins are also expressed in other tissues and secretion of apolipoproteins from liver to blood is also post-translationally regulated. For example, Apolipoprotein B is expressed in the liver, intestine and heart and its secretion can be affected by factors such as oleate and TGF-β with or without change at mRNA level^63^,^64^,^65^. Other gender-biased proteins identified are not known to be liver-enriched and not reported as differentially expressed genes in the transcriptomic study either^6^.

Combining a non-targeted, semi-quantitative shotgun MS profile and a quantitative SWATH MS profile, we have provided a comprehensive overview of zebrafish plasma proteome. 959 proteins were identified with estimated concentrations spanning almost five orders of magnitude. Based on the SWATH profile, we identified 35 gender-biased proteins, many of which were newly reported. This study has provided a valuable dataset for further studies on identification or evaluation of plasma biomarkers using zebrafish as a model.

## Methods

### Fish maintenance

This study involving zebrafish was carried out in accordance with the recommendations in the Guide for the Care and Use of Laboratory Animals of the National Institutes of Health and the protocol was approved by the Institutional Animal Care and Use Committee (IACUC) of the National University of Singapore (Protocol Number: 096/12). Adult zebrafish of 6-month old and 1.5-year old were used for testing methods of blood collection, estimation of total plasma protein content, and initial SDS-PAGE examination. For shotgun MS and SWATH, adult zebrafish (~9 months old) were purchased from a local fish farm (Mainland Fish Farm, Singapore) and allowed to acclimatize in aquaria for two weeks before sacrifice for plasma sampling. Fish maintenance and experiments were carried out following the protocols approved by Institutional Animal Care and Use Committee (IACUC) of National University of Singapore.

### Plasma sample preparation and assessment

Blood was collected using a modified tail ablation method. Briefly, fish was euthanized in ice-water and its surface was wiped dry. Tail was removed by a cut at the end of anal fin and the fish was held with the wound facing down. Whole blood was collected at the dorsal aorta using P20 micropipette fitted to an elongated tip (Prot/Elec Tips, Bio-Rad) and aspired into pre-chilled Eppendorf tubes. Both pipette tips and tubes were pre-coated with EDTA by submerging in 18 mg/mL EDTA solution for 24 hrs and then dried prior to use. Plasma was obtained as clear supernatant after centrifugation at 1000 *g* for 10 min at 4 °C. Typical yield of whole blood is 5–10 μl from male fish and 8–15 μL from female fish. Whole blood was collected to pre-chilled tubes on ice and then centrifuged within 10 min to obtain plasma. For MS analyses, each plasma sample was pooled from 9 fish. Protein concentration was determined using RCDC protein assay kit (Bio-Rad) according to the manufacturer’s protocol. For quick evaluation of protein abundance patterns, six individual plasma samples (3 males and 3 females, 0.5 μL each) were analyzed using 10% SDS gel followed by silver staining.

### Database compilation

A non-redundant *Danio rerio* protein database was compiled using databases from International protein index (40,470), UniProtKB (13,383) and NCBI GenBank (22,288) for a combined total of 76,141 non-redundant sequence entries.

### Shotgun LC-MS/MS with MASCOT search

LC-MS/MS analyses were performed in six technical replicates for each pooled sample. 2 μg of each sample from male and female was trapped on a pre-column (200 μm × 0.5 mm) and then eluted on an analytical column (75 μm × 150 mm). Both columns were packed with ChromXP C18-CL 3 μm 120 Å (Eksigent, Dublin, CA). Samples were run in a gradient formed by a mobile phase A (0.1% formic acid in 2% acetonitrile) and a mobile phase B (0.1% formic acid in 98% acetonitrile) at a flow rate of 300 nL/min. LC gradient was programmed as follows: 1 min of 95% A, 29 min of 95-88% A, 90 min of 88-70% A, 2 min of 70-10% A, 7 min of 10% A, 3 min of 10–95% and 14 min of 95% A.

Tandem MS analysis was performed using a 5600 TripleTOF analyzer (QqTOF; SCIEX) in Information Dependent Mode. Precursor ions were selected across the mass range of 400–1800 m/z using 250 ms accumulation time per spectrum. A maximum of 20 precursors per cycle from each MS spectra were selected for MS/MS analyses, with 100 ms accumulation time for each precursor and exclusion time set at 15 s. Tandem mass spectrometry was recorded in high sensitivity mode with rolling collision energy on.

The group file generated from ProteinPilot^TM^ (SCIEX) was converted to MGF peak list by its built-in converter. Protein identification was performed with MASCOT Server 2.4.0 (Matrix Science). User-defined search parameters were as follows: (1) Type of search: MS/MS Ion Search; (2) Enzyme: Trypsin; (3) Variable modifications: Acetyl (N-term), Methylthio (C) and Oxidation (M); (4) Mass Values: Monoisotopic; (5) Protein mass: Unrestricted; (6) Peptide mass tolerance: ±200 ppm; (7) Fragment mass tolerance: ±0.4 Da; (8) Max missed cleavages: 1; (9) Instrument type: ESI_QUAD-TOF; (10) Number of queries: 126,513; and (11) Taxonomy: *Danio rerio* (zebrafish). The data was searched against the compiled *Danio rerio* database (76,141 sequences) as described above with the integrated false discovery rate (FDR) analysis function. The protein identification threshold was set at 1% FDR based on reversed protein sequences. Concentrations of detected proteins were estimated using two steps: (1) the weight fraction (weight %) of each protein in plasma was obtained based on its emPAI (exponentially modified protein abundance index) score in MS profile and its molecular weight^66^; (2) the weight% was multiplied by the total plasma concentration of respective gender.

### SWATH

Ion library for SWATH was built using the same LC-MS/MS set up as in shotgun MS using 2 μg of each sample. Duplicates of male and female plasma samples were separated on the same LC gradient as described above and analyzed using a 5600 TripleTOF system (QqTOF; SCIEX,) in Information Dependent Mode. Precursor ions were selected across the mass range of 350–1250 m/z using 250 ms accumulation time per spectrum. A maximum of 20 precursors per cycle from each MS spectra were selected for MS/MS analyses with 100 ms accumulation time for each precursor and dynamic exclusion for 15 s. Tandem mass spectrometry was recorded across the mass range of 100–1800 m/z in high sensitivity mode with rolling collision energy on. Protein identification was performed with ProteinPilot™ Software 4.1 (SCIEX) as described before^67^. User-defined search parameters were as follows: (1) Sample Type: Identification; (2) Cysteine Alkylation: MMTS; (3) Digestion: Trypsin; (4) Instrument: TripleTOF5600; (5) Special Factors: None; (6) Species: *Danio rerio*; (7) ID Focus: Biological modifications; (8) Database: compiled *Danio rerio* database as described above; (9) Search Effort: Thorough; (10) the False Discovery Rate Analysis: Yes; and (11) User Modified Parameter Files: No. The proteins identified were used to generate a *SWATH*™ Ion library.

Triplicate samples from male and female fish were analyzed on a 5600 TripleTOF system (QqTOF; SCIEX,) in SWATH mode. Each acquisition method had a cycle time of 3 s to include 36 acquisition windows of 25 Da. Each acquisition window had 80 ms accumulation time and 50 ms of TOF/MS survey scan from 350 to 1250 Da. Each MS/MS acquisition window had a scan of 100 to 1,800 Da and was performed using collision energy of 35 V with a spread of 15 V. The spectra from SWATH acquisition and the ion library generated were imported into PeakView software V2.1 (SCIEX) supplemented with MS/MS (ALL) with SWATH Acquisition Microapp, version 1.0.0.653 add-on where peak alignment and data interrogation was performed. The following filters were used: (1) Peptide confidence: 99%; (2) Exclude modifications: Yes; and (3) Exclude Shared: Yes. Extracted areas were exported into MarkerView for statistical analysis. With MarkerView, a global normalization was performed on the dataset. Mean area intensity and fold change of each protein between female and male were generated and exported. Intensity values for female plasma were then multiplied by a concentration scaling factor, which was determined as the ratio of total protein concentration of female plasma over that of male plasma.

### Data analysis and biological interpretation

Gene ontology (GO) annotation for proteins detected was retrieved using Software Tool for Rapid Annotation of Proteins (STRAP, version 1.5)^68^, which extracts information from UniProt databases. Protein accessions were mapped to respective Entrez Gene IDs (GIs) and human homologous gene symbols, if available, by searching against NCBI database. Human orthologs were taken as the homologous proteins whenever available (~50%, including ~30% one-to-one ortholog pairs). When orthologs were not established, the respective homologous human genes in NCBI HomoloGene were used. Database for Annotation, Visualization and Integrated Discovery (DAVID) was used for functional annotation analysis including GO enrichment and tissue expression^69^,^70^. Ingenuity Pathway Analysis (IPA) was used for annotation and biological interpretation. For SWATH analysis, two-tailed heteroscedastic *t*-test was performed and gender-biased proteins were defined as the ones that met two criteria: *p* < 0.05 and fold change (FC) >2.

## Additional Information

**How to cite this article**: Li, C. *et al.* Comprehensive and quantitative proteomic analyses of zebrafish plasma reveals conserved protein profiles between genders and between zebrafish and human. *Sci. Rep.* **6**, 24329; doi: 10.1038/srep24329 (2016).

## Supplementary Material

Supplementary Table S1

Supplementary Table S2

Supplementary Table S5

Supplementary Figures and Supplementary Tables S3, S4 and S6

## Figures and Tables

**Table 1:** Top 30 abundant proteins in female and male zebrafish plasma.

Female			Male		
Protein Name	Gene Symbol	Estimated Conc. (mg/mL)	Protein Name	Gene Symbol	Estimated Conc. (mg/mL)
*Apolipoprotein A-Ib precursor	*apoa1b*	8.60	*Apolipoprotein A-Ib precursor	*apoa1b*	7.43
Vitellogenin 4	*vtg4*	4.51	*Apolipoprotein A-I	*apoa1*	2.30
Vitellogenin 6	*vtg6*	4.14	*PREDICTED: serotransferrin isoform X2	*tfa*	1.89
Vitellogenin 5	*vtg5*	3.82	*Hemopexin	*hpx*	1.85
Vitellogenin 1	*vtg1*	3.73	*Complement component c3a, duplicate 2 precursor	*c3a.2*	1.36
Vitellogenin 7 precursor	*vtg7*	2.69	*Hemoglobin subunit alpha	*hbaa1*	0.80
Vitellogenin 2	*vtg2*	2.52	*Uncharacterized protein (apolipoprotein Bb, tandem duplicate 1)	*apobb.1*	0.76
*Apolipoprotein A-I	*apoa1*	1.74	*Complement component c3a precursor	*c3a.1*	0.73
*Hemopexin	*hpx*	0.96	Alpha-2-macroglobulin-like isoform 1	*LOC100006895*	0.73
*PREDICTED: serotransferrin isoform X2	*tfa*	0.95	*Apolipoprotein A-IV-like precursor	*apoa4b.2*	0.61
*Uncharacterized protein (PREDICTED: apolipoprotein Bb, tandem duplicate 1 isoform X1)	*apobb.1*	0.88	*PREDICTED: apolipoprotein B-100	*LOC100330435*	0.59
*Apolipoprotein A-IV-like precursor	*apoa4b.2*	0.79	*Hemoglobin subunit beta-2	*ba2*	0.57
*Complement component c3a, duplicate 2 precursor	*c3a.2*	0.58	*Hemoglobin subunit beta-1	*ba1*	0.57
*PREDICTED: apolipoprotein A-I-like	*apoa4b.3*	0.57	Ceruloplasmin	*cp*	0.50
*Apolipoprotein A-IV	*apoa4b.1*	0.51	*Complement component C3-2	*c3a.3*	0.50
*Complement component c3a precursor	*c3a.1*	0.45	*Apolipoprotein A-IV	*apoa4b.1*	0.49
*Complement component C3-2	*c3a.3*	0.43	*Fibrinogen, B beta polypeptide	*fgb*	0.47
Vitellogenin 3	*vtg3*	0.42	Uncharacterized protein LOC322327 precursor (Apolipoprotein A-II)	*apoa2*	0.45
*Hemoglobin subunit alpha	*hbaa1*	0.39	*PREDICTED: apolipoprotein A-I-like	*apoa4b.3*	0.44
*Fibrinogen, B beta polypeptide	*fgb*	0.37	*Fibrinogen, alpha polypeptide	*fga*	0.36
*PREDICTED: apolipoprotein B-100	*LOC100330435*	0.37	Uncharacterized protein (Fibronectin)	*fn1b*	0.31
Apolipoprotein Eb	*apoeb*	0.35	*Uncharacterized protein (PREDICTED: apolipoprotein B-100)	*apoba*	0.31
*Hemoglobin subunit beta-2	*ba2*	0.33	*Serpina1 protein like	*serpina1l*	0.31
*Hemoglobin subunit beta-1	*ba1*	0.33	*Alpha-2-macroglobulin	*sb:cb37*	0.29
*Uncharacterized protein (PREDICTED: apolipoprotein B-100)	*apoba*	0.29	*Serpina1 protein (Fragment)	*serpina1*	0.28
*Fibrinogen, alpha polypeptide	*fga*	0.28	Immunoglobulin heavy constant mu	*ighm*	0.28
*Serpina1 protein like	*serpina1l*	0.26	*Alpha-2-macroglobulin-like precursor	*a2ml*	0.28
*Alpha-2-macroglobulin	*sb:cb37*	0.26	Fibrinogen, gamma polypeptide	*fgg*	0.24
*Serpina1 protein (Fragment)	*serpina1*	0.25	PREDICTED: alpha-2-macroglobulin-like	*si:dkey-105h12.2*	0.23
*Alpha-2-macroglobulin-like precursor	*a2ml*	0.24	Complement component 5	*c5*	0.22

Some entries were labeled as “uncharacterized” in protein database but unambiguously mapped to a known zebrafish gene. The gene name was given in brackets in such cases.

*Proteins that are among top 30 abundant ones in both genders.

**Table 2:** Gender-biased proteins (*p* < 0.05, fold change >2 or <−2) in zebrafish plasma.

Protein	Gene Symbol	Entrez GI	HUMAN Homolog	Fold change
**Female-biased**				**(F/M)**
Vitellogenin 1	vtg1	559475		1552.54
Vitellogenin 2 (Q1MTC4)	vtg2	559931		414.70
Vitellogenin 7 precursor	vtg7	559856		218.59
Vitellogenin 6	vtg6	559229		186.10
Vitellogenin 5	vtg5	64260		96.13
Vitellogenin 4	vtg4	678536		73.91
Vitellogenin 3	vtg3	30518		30.95
Uncharacterized protein (Coagulation factor XIII)	f13a1a.1	767742		23.16
Uncharacterized protein (Nothepsin)	nots	114367		22.64
Vitellogenin 2 (A8WGJ1)	vtg2	559931		19.73
Vitelline membrane outer layer 1 homolog a	vmo1a	793369	VMO1	6.38
Apolipoprotein Eb	apoeb	30314	APOE	4.19
Phosphoglycerate kinase 1	pgk1	406696	PGK1	4.16
PREDICTED: saxitoxin and tetrodotoxin-binding protein 2-like	LOC795897	795897		4.02
Branched chain keto acid dehydrogenase E1, beta polypeptide, like	bckdhbl	555648	BCKDHB	3.95
PREDICTED: keratin, type II cytoskeletal 8 isoform X1	krt8	797433	KRT8	3.75
Apolipoprotein E precursor	apoea	553587	APOE	3.46
Ckmb protein (Muscle creatine kinase b)	ckmb	794752	CKM	3.33
Triosephosphate isomerase B	tpi1b	560753		3.02
PREDICTED: apolipoprotein Bb, tandem duplicate 1 isoform X1	apobb.1	321166	APOB	2.65
Nucleoside diphosphate kinase	nme2b.2	30084	NME2	2.48
Muscle creatine kinase a	ckma	30095	CKM	2.37
Retinol binding protein 4, plasma	rbp4	30077	RBP4	2.18
Apolipoprotein C-II	apoc2	568972	APOC2	2.15
Apolipoprotein A-IV	apoa4b.1	322543	APOA4	2.12
Uncharacterized protein (Parvalbumin 1)	pvalb1	402805		2.08
**Male-biased**				**(M/F)**
Uncharacterized (Carboxylesterase 2-like)	ces2	566132	CES1	3.59
Ependymin	epd	30199		3.39
Serpina1 protein like	serpina1l	321195	SERPINA1	3.13
Uncharaterized protein (si:ch1073-126c3.2)	si:ch1073-126c3.2	555816		3.12
Uncharacterized protein LOC791587	zgc:174259	791587	SERPINA9	2.53
Hypothetical protein LOC334459	wu:fi74c02	334459		2.25
Sex hormone binding globulin	shbg	322604	SHBG	2.21
PREDICTED: complement C4-B	c4	566261	C4A/C4B	2.09
Myoglobin	mb	393558	MB	2.05
