# Supplementary material for: Comprehensive and quantitative proteomic analyses of zebrafish plasma reveals conserved protein profiles between genders and between zebrafish and human
Source: Sci Rep. 2016 Apr 13;6:24329. doi: 10.1038/srep24329 (PMC4829857; doi:10.1038/srep24329)
Supplement: Supplementary Figures and Supplementary Tables S3, S4 and S6 [file srep24329-s4.pdf]

Supplementary Information

Comprehensive and quantitative proteomic analyses of zebrafish plasma reveals conserved protein profiles between genders and between zebrafish and human

Caixia Li, Xing Fei Tan, Teck Kwang Lim, Qingsong Lin*, Zhiyuan Gong*

Department of Biological Sciences, National University of Singapore, Singapore 117543

*Authors for correspondence

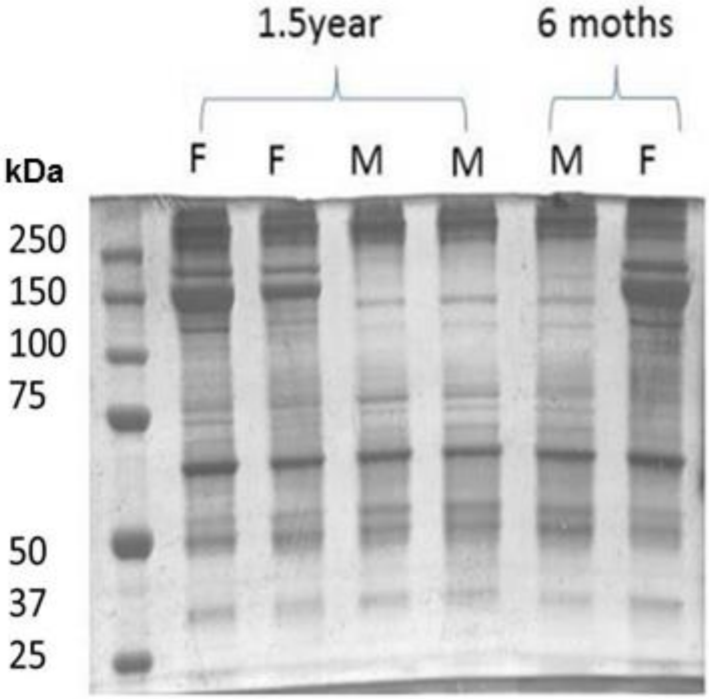

Supplementary Figure S1. SDS polyacrylamide gel electrophoresis pattern of plasma proteins after silver staining. Six individual fish of both female (F) and male (M) of 6 month old or 1.5 year old are shown and for each lane, 0.5 µL of plasma was loaded.

A

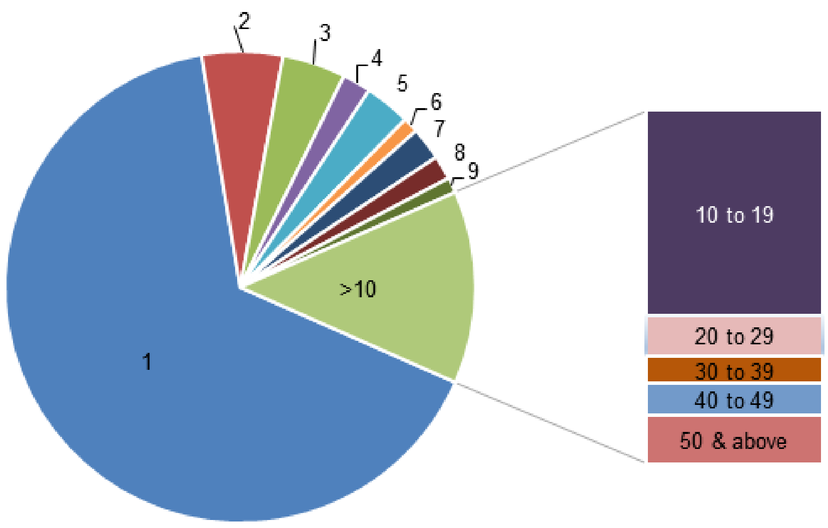

B

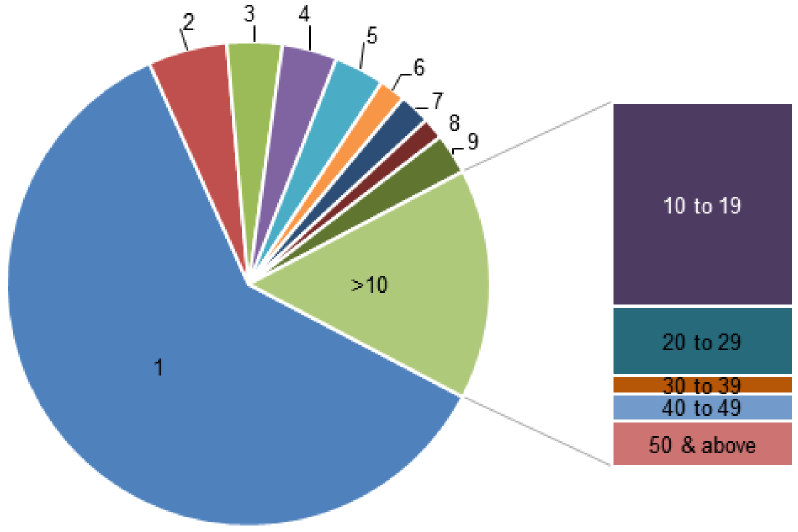

Supplementary Figure S2. Distribution plot of numbers of significant peptides per protein identified in female (A) and male (B). About 40% of the proteins were identified with two or more significant peptides.

A

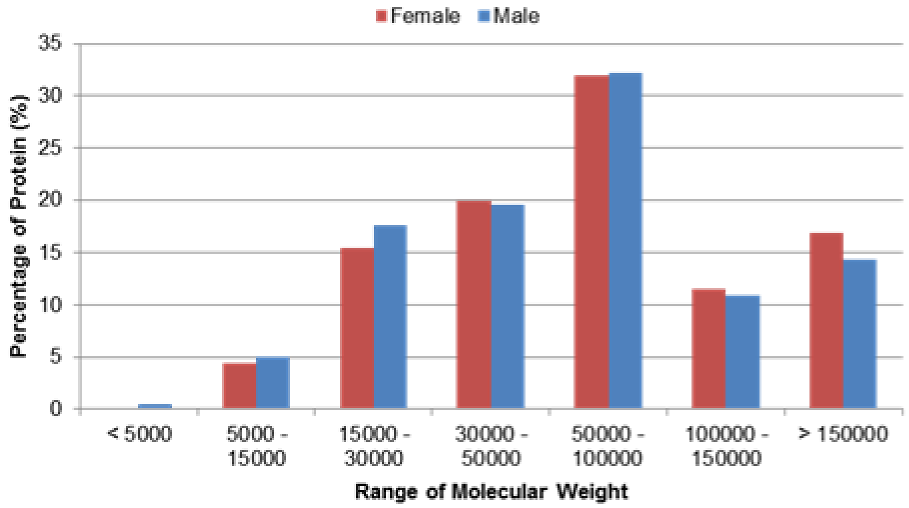

B

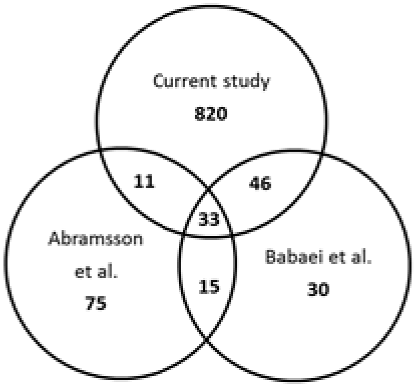

Supplementary Figure S3. Shotgun profile of zebrafish plasma proteome. (A) Distribution of female and male plasma proteins based on molecular weights. (B) Venn diagram of proteins detected in current shotgun profile and two previous studies[17,28]. Proteins were converted to gene IDs for direct comparison using Venny version 2.0 (http://bioinfogp.cnb.csic.es/tools/venny/).

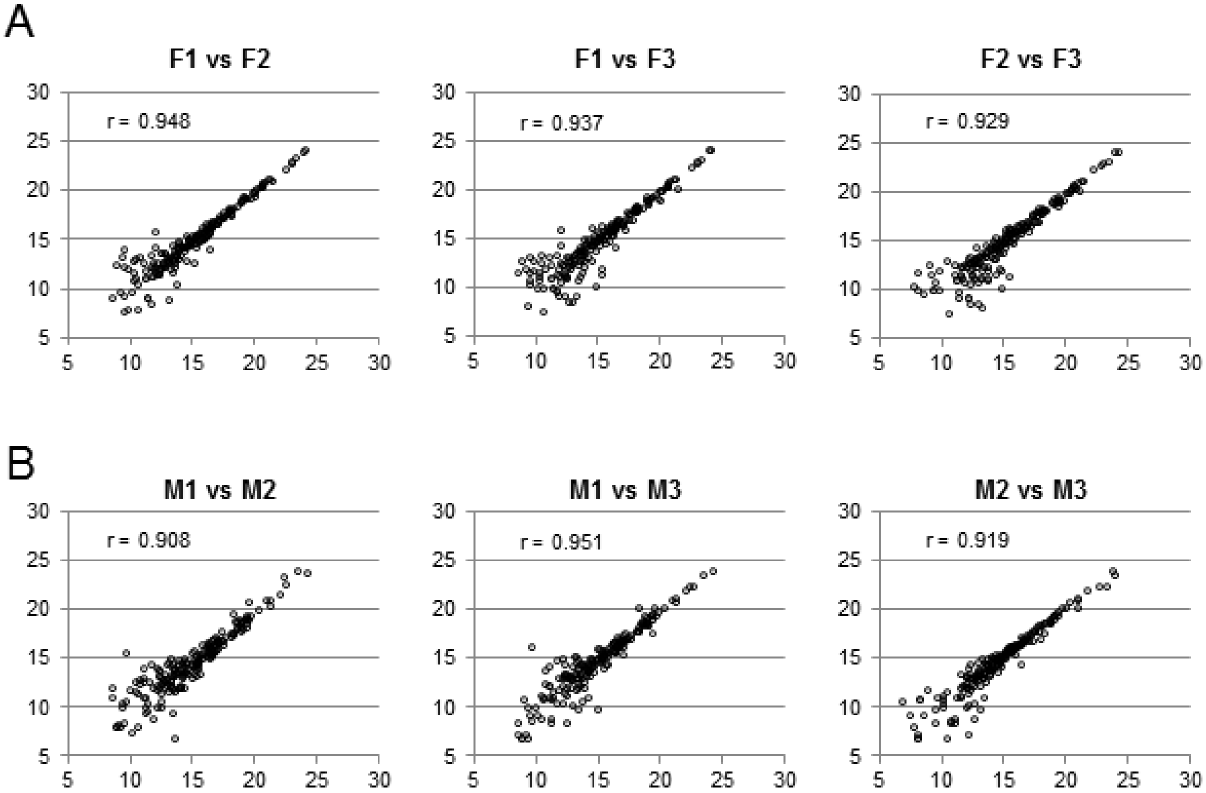

Supplementary Figure S4. Quality assessment of SWATH based on Pearson correlation between replicates. Log2 transformation correlation plot of three SWATH runs for female replicates (A) and male replicates (B) show good correlation between replicates except slightly higher variation in proteins with lower abundance.

Supplementary Table S1. List of 642 unique proteins detected in female plasma using shotgun profiling (xls file).

Supplementary Table S2. List of 594 unique proteins detected in male plasma using shotgun profiling (xls file).

Supplementary Table S3. Types of plasma proteins detected in two genders. Information for a total of 518 genes was retrieved in IPA.

**Types of proteins**	**Number**	**Number in female**	**Weight% in female**	**Number in male**	**Weight% in male**
Enzyme	98	69	1.716	60	3.917
Transporter	47	42	33.050	31	54.218
Peptidase	37	26	3.934	33	9.560
Kinase	29	19	0.823	14	1.071
Transcription regulator	25	18	0.095	18	0.065
Transmembrane receptor	9	7	0.055	5	0.063
Ion channel	9	6	0.034	4	0.025
Translation regulator	7	5	0.034	5	0.012
Growth factor	2	1	0.074	2	0.151
G-protein coupled receptor	8	5	0.028	5	0.019
Phosphatase	3	2	0.010	3	0.020
Cytokine	2	2	0.294	2	0.648
Other	242	152	5.117	151	9.540

Supplementary Table S4. Tissue expression of genes coding for the plasma proteins detected. Information for 452 genes was retrieved using DAVID.

	Mapped to one term only	Mapped to Multiple Terms	Number in Female	Sum of weight% in Female	Number in male	Sum of weight% in male
Liver	37	32	56	34.21	56	26.26
Kidney	31	31	47	1.08	40	2.05
Olfactory epithelium	21	19	27	0.08	30	0.13
Eye	11	6	10	0.03	11	0.04
Ovary	9	16	17	0.03	17	0.05
Brain	8	8	10	0.02	13	0.07
Gut	6	7	11	0.28	12	0.39
Heart	6	7	8	0.16	9	0.77
Testis	3	2	4	0.01	4	0.02
Gill	2	4	6	0.05	5	0.05
Skin	1	7	6	0.03	5	0.06
Pooled/Mixed Tissues	25	25	35	0.70	33	1.57
Whole body	92	66	122	5.07	112	10.22
Larvae	8	11	15	0.05	14	0.17
Embryo	64	58	74	1.54	76	2.37

Supplementary Table S5. List of 200 proteins quantified using SWATH. TIC values have been global-normalized and adjusted by total plasma concentration (xls file).

Supplementary Table S6. Validation of SWATH-identified gender-biased proteins in shotgun profile.

		Fold change (F/M) in SWATH	Concentration (μg/mL) in female shotgun profile	Concentration (μg/mL) in male shotgun profile
Female biased proteins	**26**			
**Absent in male shotgun profille**	**10**			
Vitellogenin 1		1552.54	3727.52	
Vitellogenin 2		414.70	2516.28	
Vitellogenin 7		218.59	2691.59	
Vitellogenin 6		186.10	4143.02	
Vitellogenin 5		96.13	3822.74	
Vitellogenin 4		73.91	4513.20	
Vitellogenin 3		30.95	421.87	
Coagulation factor XIII		23.16	52.15	
Nothepsin		22.64	7.76	
Vitellogenin 2		19.73	2516.28	
**F>M in concentration**	**10**			
Apolipoprotein Eb		4.19	353.09	157.35
PREDICTED: saxitoxin and tetrodotoxin-binding protein 2-like		4.02	21.80	20.94
Branched chain keto acid dehydrogenase E1, beta polypeptide, like		3.95	2.44	2.34
Apolipoprotein E precursor		3.46	79.13	22.29
Muscle creatine kinase b		3.33	116.71	83.78
PREDICTED: apolipoprotein Bb, tandem duplicate 1 isoform X1		2.65	883.92	755.00
Nucleoside diphosphate kinase		2.48	149.09	131.18
Retinol binding protein 4, plasma		2.18	71.62	39.59
Apolipoprotein C-II		2.15	44.71	31.07
Apolipoprotein A-IV		2.12	510.26	490.12
**F<M in concentration**	**4**			
Phosphoglycerate kinase 1		4.16	4.86	7.46
Triosephosphate isomerase B		3.02	15.55	28.93
Muscle creatine kinase a		2.37	87.46	102.18
Uncharacterized protein (Parvalbumin 1)		2.08	9.93	21.71
**Absent in female shotgun profile**	**2**			
Vitelline membrane outer layer 1 homolog a		6.38		2.35
PREDICTED: keratin, type II cytoskeletal 8 isoform X1		3.75		2.41

Male biased proteins	**9**			
**Absent in female shotgun profille**	**1**			
Ependymin		0.29		11.42
**M>F in concentration**	**7**			
Myoglobin		0.49	5.42	41.85
PREDICTED: complement C4-B		0.48	27.68	107.67
Sex hormone binding globulin		0.45	57.29	164.51
Hypothetical protein LOC334459		0.44	5.29	12.25
Uncharaterized protein (si:ch1073-126c3.2)		0.32	5.20	15.17
Serpina1 protein like		0.32	258.06	305.09
Uncharacterized (Carboxylesterase 2-like)		0.28	126.05	177.19
**M<F in concentration**	**0**			
**Absent in male shotgun profile**	**1**			
Uncharacterized protein LOC791587		0.39	45.03	
